# The Interplay Between Hyperthyroidism and Ovarian Cytoarchitecture in Albino Rats

**DOI:** 10.7759/cureus.14517

**Published:** 2021-04-16

**Authors:** Tayyaba Mahmud, Qudsia U Khan, Sarah Saad

**Affiliations:** 1 Anatomy, Combined Military Hospital (CMH) Lahore Medical College and Institute of Dentistry, Lahore, PAK; 2 Physiology, Combined Military Hospital (CMH) Lahore Medical College and Institute of Dentistry, Lahore, PAK

**Keywords:** hyperthyroidism, ovarian follicle, fertility, wistar rats

## Abstract

Background

Hyperthyroid females often complain of menstrual disturbances and impaired fertility. This study was designed to observe the effect of hyperthyroidism on ovarian folliculogenesis and the hypophyseal-gonadal axis.

Methodology

Adult female Wistar albino rats (n= 12), six to eight weeks of age, and weighing 70-162 g, were divided randomly into control (Group A) and experimental (Group B) groups. Group A received daily intraperitoneal injections of 250 µL normal saline (10 µL 5 µM NaOH dissolved in it) for 14 days. Group B received a daily intraperitoneal injection of levothyroxine (600 µg/kg body weight) to induce hyperthyroidism. Rats were weighed at the start and the end of the experimental period on the day of sacrifice.

Results

Statistical analysis of the data revealed successful induction of hyperthyroidism in Group B as their thyroid-stimulating hormone (TSH) levels decreased significantly. The ovarian size was significantly reduced in the hyperthyroid group (p < 0.029). There was a significant decrease in thickness of the ovarian capsule (p < 0.000), an increase in the number of primordial, primary, and secondary follicles (p < 0.001, 0.000, and 0.001, respectively), and a decrease in size of primary and secondary follicles (p < 0.041 and 0.020) in the hyperthyroid group.

Conclusion

Hyperthyroidism can affect ovarian cytoarchitecture, probably by acting directly on its receptors and thus affects female fertility.

## Introduction

For normal growth and development, the thyroid hormones (TH) regulate the basal metabolic rate and play a crucial role in the development of multiple organ systems including the reproductive system [1]. Thyroid hormones are essential for the female reproductive system due to their involvement in the function of ovaries. Thyroid hormone receptors are generated by oocytes, granulosa, and cumulus cells, meaning that thyroid hormones may act upon and thus influence ovarian tissue [2]. For normal follicular development, equilibrium in the output and work of hormones in the hypothalamus-pituitary-ovarian axis and hypothalamic-pituitary-thyroid axis is necessary. T3 specifically stimulates the development of ovarian granulosa cells, acting synergistically with follicle-stimulating hormone (FSH) to inhibit apoptosis in rats [3]. The importance of thyroid hormones in the development of preantral and antral follicles in the ovary is already reported [4].

Elevated levels of thyroid hormones in the blood can influence the function and development of all main organs [5]. It is suggested that mean circulating levels of thyroxine, triiodothyronine, and thyroid-stimulating hormone can influence the functionality of the ovarian reserve determining infertility [6]. Hyperthyroid females often complain of menstrual disturbances and fertility impairment. Their symptoms are resolved by restoring a normal thyroid status [7].

In laboratory studies, low levels of the female hormone estradiol and progesterone have been recorded in adult hyperthyroid rats [8]. Similarly, the synthesis of progesterone was reported to enhance in the hyperthyroid condition due to progesterone stimulating factor activity, which subsequently increased the number of ovarian follicles [9]. In some studies, the amounts of FSH and luteinizing hormone (LH) were either unchanged or decreased in a hyperthyroid state [10].

Hyperthyroidism can contribute to subfertility or infertility in both human and animal females [11]. Thyroid hormone dysregulation may inhibit ovarian follicular production. Glucose-regulated protein 78 (GRP78), an antiapoptotic master regulator for endoplasmic reticulum stress, has been identified as a novel mediator of TH. The GRP78 expression profile is altered by TH dysregulation, suggesting that TH dysregulation can stimulate the apoptotic signaling cascade. This in turn can trigger ovarian cell apoptosis [12]. A study uncovered a connection between the thyroid hormone and nitric oxide signaling pathways at the time of ovarian follicle development in immature rats [13].

T3, normally, acts as a tumor suppressor due to its ability to promote cell differentiation. However, hyperthyroidism and ovarian cancer have been associated more recently, and it has been observed that hyperthyroidism increases levels of deiodinase type 3, which then promotes cancer proliferation. Deiodinase type 3 has been found in high-grade serous ovarian cancer (HGSOC) samples of human patients [14]. The effect of thyroid-stimulating hormone (TSH) on induced egg development may have been altered by the thyroid-stimulating hormone receptor/cyclic AMP (TSHR/cAMP), TSHR expression level signaling cascade in granulosa cells [15].

Considering that thyroid dysfunction can lead to a multitude of conditions, the current study was designed to investigate the morphological changes taking place in the ovaries of hyperthyroid rats. In addition, it was decided to relate any potential change in cytoarchitecture to possible variations in the sex hormones (estradiol and progesterone) and gonadotropins (FSH and LH).

## Materials and methods

It was an experimental study that was performed under the declaration of the World Medical Association (WMA) made in Helsinki (2008) regarding the ethical principles for medical research involving experimental animals. The study was approved by the institutional ethical review committee (the ethical approval number was not provided by the institution).

Adult female Wistar albino rats (n= 12), six to eight weeks of age and weighing 70-162 g were raised in the animal house of the University of Health Sciences, Lahore, Pakistan. These rats were grouped randomly into control (Group A) and experimental (Group B). Standard laboratory conditions were maintained for the rats at controlled temperature (22 ± 2°C) and humidity (55% ± 5%), with a light and dark cycle of 12 hours each. Rats were fed on standard rat chow and water *ad libitum*.

Group A received daily intraperitoneal injections of 250 µL normal saline, with 10 µL 5 µM NaOH dissolved in it, for two weeks. To induce hyperthyroid state, rats of Group B received a daily intraperitoneal injection of levothyroxine (600 µg/kg body weight) which was dissolved in 250 µL normal saline (containing 10 µL 5 µM NaOH), for two weeks [16].

At the end of the experimental period, the estrous phase of the rat cycle was ascertained by examining the vaginal smear under the microscope. Rats were anesthetized with chloroform. Blood was collected from each animals’ heart to determine the serum levels of TSH, FSH, LH, estradiol, and progesterone. TSH, FSH, and LH were quantified using enzyme-linked immunosorbent assay (ELISA) kits (Elabscience Co., Houston, TX) and estradiol and progesterone were quantified using ELISA kits (BioCheck, Inc. Foster City, CA) according to the manufacturer’s protocol.

The abdominal cavity was opened using midline vertical and transverse incisions to expose ovaries. Ovaries were then removed and fixed in 10% formalin. The size of the formalin-fixed ovaries was measured using a vernier caliper. Serial sections of ovaries (5 µm) were prepared. The thickness of tunica albuginea of ovary, size, and the number of follicles, containing the nucleus of an oocyte, were measured in the large cross-sections (LCS) in 12 sections by micrometry [17]. The thickness of tunica albuginea was measured in slides stained with Masson’s trichrome stain. The number and size of primordial, primary, secondary, and Graafian follicles were taken into account in slides stained with hematoxylin and eosin.

Data analysis was done using Statistical Package for Social Sciences (SPSS) Version 20.0 (IBM Corp, Armonk, NY). Shapiro-Wilk test was used to check the distribution of the data obtained. Independent samples t-test was applied for the analysis of normally distributed data, while analysis of non-normally distributed data was done by applying Mann Whitney U test. P-value < 0.05 was considered statistically significant.

## Results

**Figure 1:**
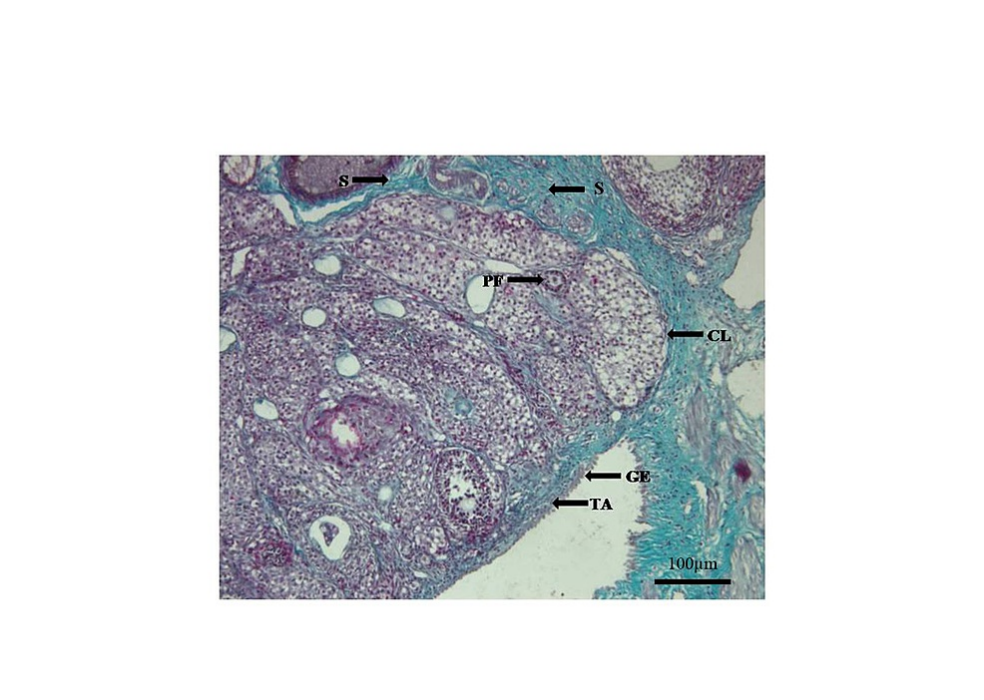
Photomicrograph of the ovary from Group A (control), showing stromal components of the ovary. Masson’s trichrome. x100 Germinal epithelium (GE), tunica albuginea (TA), the primary follicle (PF), corpus luteum (CL), stroma (S)

**Figure 2:**
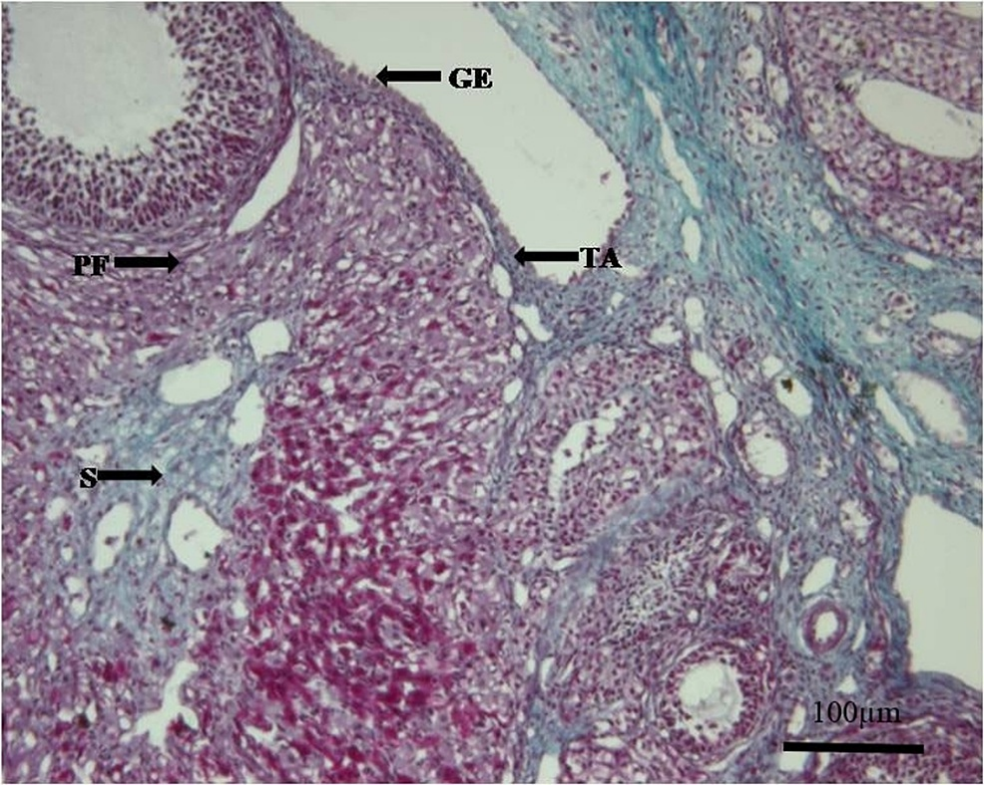
Photomicrograph of the ovary from Group B (experimental). Thinning of the capsule/TA of the ovary can be seen. Masson’s trichrome. x100 Tunica albuginea (TA), germinal epithelium (GE), the primary follicle (PF), stroma (S)

Statistical analysis of the data showed that experimental hyperthyroid state was successfully induced into rats of Group B as their TSH levels decreased considerably in response to levothyroxine administration. There was a statistically insignificant change in the levels of LH, FSH, estradiol, and progesterone when compared among control and experimental groups. However, the ovarian size and thickness of the ovarian capsule in hyperthyroid rats decreased significantly (Tables 1, 2 and Figures 1, 2).

**Table 1:** Mean ± SD for different types of hormones when compared between Group A and Group B *P-value < 0.05 Thyroid-stimulating hormone (TSH), follicle-stimulating hormone (FSH), luteinizing hormone (LH)

Parameters	Group A	Group B	P-value
Serum TSH (ng/mL)	3.35 ± 5.06	0.88 ± 0.63	0.02*
Serum LH (mIU/mL)	6.33 ± 5.01	7.99 ± 1.71	0.88
Serum FSH (ng/mL)	5.07 ± 2.52	5.95 ± 1.74	0.10
Serum estradiol (pg/mL)	5.13 ± 2.01	5.14 ± 2.19	0.99
Serum progesterone (ng/mL)	11.53 ± 8.77	11.51 ± 2.90	0.24

**Table 2:** Comparison of mean values of different ovarian parameters between control (A) and hyperthyroid (B) groups *P-value < 0.05

Parameters		Group A	Group B	P-value
		Mean ± SD	Mean ± SD	
Size of ovaries (mm)		6.55 ± 0.71	5.77 ± 0.48	0.029*
The thickness of tunica albuginea (µm)		17.75 ± 3.81	8.00 ± 1.55	0.000*
Primordial follicles	Number/mm^2^	2.77 ± 0.74	4.97 ± 1.60	0.001*
	Size (µm)	18.00 ± 2.52	17.32 ± 2.43	0.443
Primary follicles	Number/mm^2^	1.85 ± 0.72	3.14 ± 0.52	0.000*
	Size (µm)	65.23 ± 13.22	54.87 ± 9.67	0.041*
Secondary follicles	Number/mm^2^	1.26 ± 0.28	1.69 ± 0.24	0.001*
	Size (µm)	206.32 ± 31.73	183.61 ± 13.63	0.020*
Graafian follicles	Number/mm^2^	1.00 ± 0.00	1.11 ± 0.17	0.053
	Size (µm)	438.10 ± 84.04	427.92 ± 69.21	0.749

**Figure 3:**
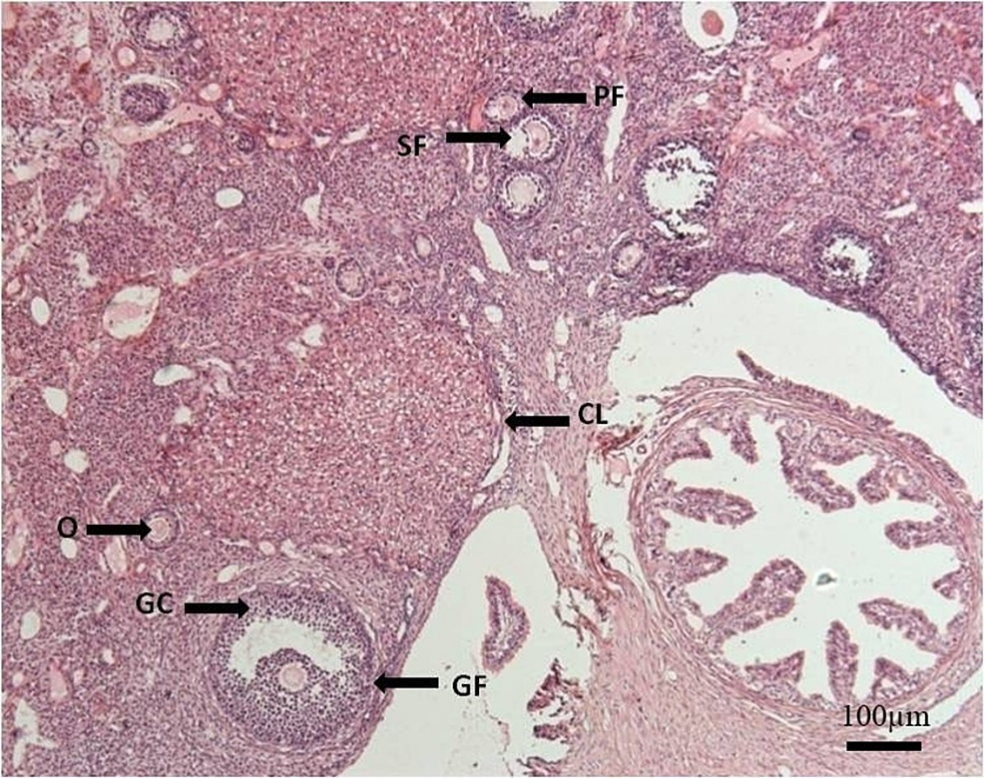
Photomicrograph of the ovary from Group A (control), showing different stages of follicular development. H&E. x50 Primary follicle (PF), the secondary follicle (SF), Graafian follicle (GF), corpus luteum (CL), granulosa cells (GC), oocyte (O), hematoxylin and eosin (H&E)

**Figure 4:**
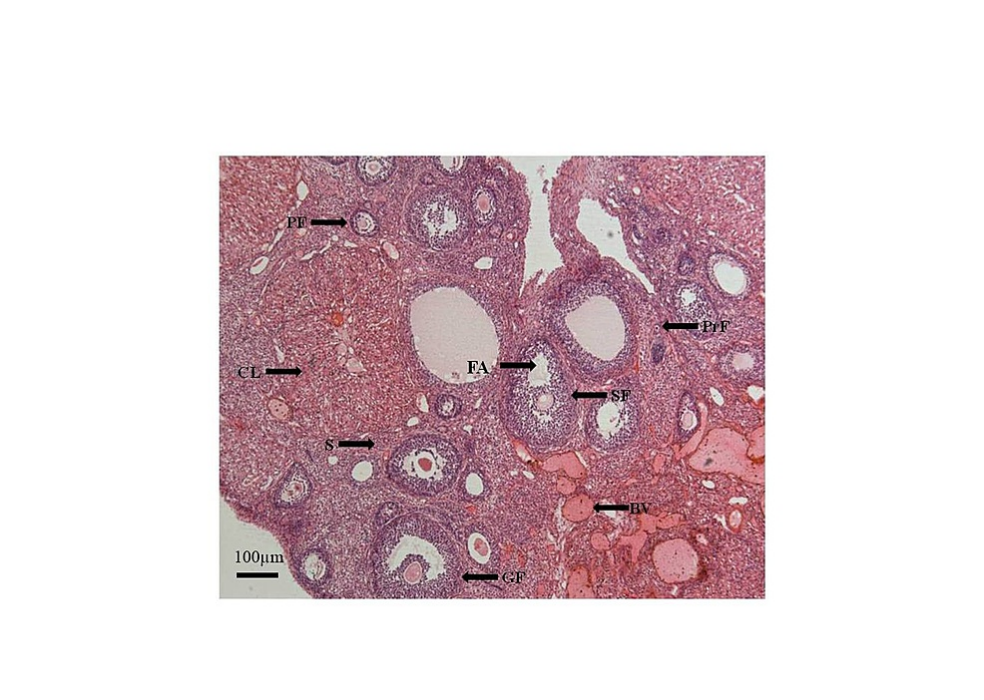
Photomicrograph of the ovary from Group B (experimental), showing an increased number of follicles. H&E. x50 Primordial follicle (PrF), the primary follicle (PF), the secondary follicle (SF), Graafian follicle (GF), corpus luteum (CL), stroma (S), follicular antrum (FA), blood vessel (BV), hematoxylin and eosin (H&E)

There was a statistically significant increase in follicular count (primordial, primary, and secondary follicles) in the ovaries of hyperthyroid rats. This was accompanied by a statistically significant decrease in the size of only primary and secondary follicles in the ovaries of the experimental group (Table 2 and Figures 3, 4).

## Discussion

In recent times, multiple studies have been conducted to observe the effect of thyroid dysfunction on the reproductive system [18]. This study was designed to observe the effect of experimentally induced hyperthyroid state on ovarian parameters and hypophyseal-gonadal axis, in adult Wistar albino rats.

In this study, hyperthyroidism caused a decrease in ovarian size, accompanied by an increased number of ovarian follicles. A similar increase in the ovarian follicular number was reported previously [19,20] though another group of researchers reported a decline in the ovarian follicular count in the hyperthyroid state [21]. Triiodothyronine acts synergistically with FSH to inhibit the apoptosis of granulosa cells and stimulate cellular proliferation [13]. However, in the current study, the rise in the number of ovarian follicles was independent of any significant change in gonadal hormone levels. The numerical increase, observed in serum levels of FSH and LH in hyperthyroid rats of this study, was not statistically significant, as observed in other studies as well [22,23]. The presence of THRs on the ovary is already documented in the literature [24]. Thus, it is probable that the number of ovarian follicles increased in response to the direct effect of THs on THRs present on the ovaries. In addition, it is also hypothesized that TH can increase the expression of the neural apoptosis inhibitory protein (NAIP) gene independent of gonadotropins. NAIP gene is expressed in ovarian follicles undergoing different stages of development, except atretic follicles. Increased gonadotropin levels usually increase the NAIP gene expression in ovaries, resulting in delayed apoptosis of granulosa cells, thereby leading to an increase in the number of growing, viable follicles [25]. Further work is required to establish the role of thyroid hormones, with or without the interplay of gonadotropins, on the expression of the NAIP gene.

The increased follicular number appeared to produce a pressure effect on the ovarian stroma. This, along with an increase in lytic effect due to hyperthyroidism, caused a decrease in ovarian stroma. The increasing number of follicles within the boundaries of tunica albuginea also compromised the corresponding increase in the size of follicles, thus leading to a significant decrease in the size of primary and secondary follicles.

A statistically insignificant change was observed in estradiol levels of hyperthyroid rats. This might be due to the suppression of FSH-induced action of aromatase, caused by THs. Some studies documented similar results, while a few others reported contradictory results [5,14]. The definite mechanism by which thyroid hormones regulate gonadotropins is not well-established. Concisely, it can be deduced that raised levels of thyroid hormones directly act on ovaries to stimulate folliculogenesis, even without the action of FSH.

This study did not use special staining techniques to stain thyroid hormone receptors in ovaries of both control and hyperthyroid rats. Further work can be done in the future to ascertain any change in the expression of different types of thyroid hormone receptors on the ovary in a hyperthyroid state. This may help in clarifying if hyperthyroid state affects ovarian morphology by causing overexpression or underexpression of THRs.

## Conclusions

It can be concluded that hyperthyroid state produces changes in ovarian cytoarchitecture, independent of the hypophyseal-gonadal axis. Thyroid dysfunction can affect ovarian morphology, possibly by acting directly on its receptors. This can bring about significant morphological changes in the ovary, and thus, can affect female fertility even in the absence of any abnormality in the hormonal profile of the affected individual.
